# Trends in Laboratory-Confirmed SARS-CoV-2 Reinfections and Associated Hospitalizations and Deaths Among Adults Aged ≥18 Years — 18 U.S. Jurisdictions, September 2021–December 2022

**DOI:** 10.15585/mmwr.mm7225a3

**Published:** 2023-06-23

**Authors:** Kevin C. Ma, Vajeera Dorabawila, Tomás M. León, Hannah Henry, Amelia G. Johnson, Eli Rosenberg, Joshua A. Mansfield, Claire M. Midgley, Ian D. Plumb, Julia Aiken, Quratul Ain Khanani, Steven Auche, Nagla S. Bayoumi, Sarah A. Bennett, Carmen Bernu, Carolyn Chang, Kathryn J. Como-Sabetti, Kevin Cueto, Spencer Cunningham, Meredith Eddy, Rebecca A. Falender, Aaron Fleischauer, Darren M. Frank, Pauline Harrington, Mikhail Hoskins, Adam Howsare, Lucy M. Ingaiza, Aras S. Islam, Shelli A. Jensen, Jefferson M. Jones, Grace Kambach, FNU Kanishka, Yuriy Levin, John F. Masarik, Stephanie D. Meyer, Lauren Milroy, Keeley J. Morris, John Olmstead, Nina S. Olsen, Enaholo Omoike, Komal Patel, Amanda Pettinger, Melissa A. Pike, Isaiah G. Reed, Elizabeth Slocum, Melissa Sutton, Buddhi P. Tilakaratne, Hailey Vest, Johanna Vostok, Jennifer S. Wang, Lydia Watson-Lewis, Haley N. Wienkes, Melissa Briggs Hagen, Benjamin J. Silk, Heather M. Scobie

**Affiliations:** ^1^Coronavirus and Other Respiratory Viruses Division, National Center for Immunization and Respiratory Diseases, CDC; ^2^Epidemic Intelligence Service, CDC; ^3^New York State Department of Health; ^4^California Department of Public Health; ^5^Louisiana Department of Health; ^6^Colorado Department of Public Health and Environment; ^7^Washington State Department of Health; ^8^Kentucky Department for Public Health; ^9^New Jersey Department of Health; ^10^Indiana Department of Health; ^11^Minnesota Department of Health; ^12^New York City Department of Health and Mental Hygiene, New York, New York; ^13^Nebraska Department of Health and Human Services; ^14^Massachusetts Department of Public Health; ^15^Oregon Health Authority; ^16^North Carolina Department of Health and Human Services; ^17^Michigan Department of Health and Human Services; ^18^Philadelphia Department of Public Health, Philadelphia, Pennsylvania; ^19^Tennessee Department of Health; ^20^District of Columbia Department of Health, Washington, DC; ^21^Georgia Department of Public Health; ^22^CDC Foundation, Atlanta, Georgia.

Although reinfections with SARS-CoV-2 have occurred in the United States with increasing frequency, U.S. epidemiologic trends in reinfections and associated severe outcomes have not been characterized. Weekly counts of SARS-CoV-2 reinfections, total infections, and associated hospitalizations and deaths reported by 18 U.S. jurisdictions during September 5, 2021–December 31, 2022, were analyzed overall, by age group, and by five periods of SARS-CoV-2 variant predominance (Delta and Omicron [BA.1, BA.2, BA.4/BA.5, and BQ.1/BQ.1.1]). Among reported reinfections, weekly trends in the median intervals between infections and frequencies of predominant variants during previous infections were calculated. As a percentage of all infections, reinfections increased substantially from the Delta (2.7%) to the Omicron BQ.1/BQ.1.1 (28.8%) periods; during the same periods, increases in the percentages of reinfections among COVID-19–associated hospitalizations (from 1.9% [Delta] to 17.0% [Omicron BQ.1/BQ.1.1]) and deaths (from 1.2% [Delta] to 12.3% [Omicron BQ.1/BQ.1.1]) were also substantial. Percentages of all COVID-19 cases, hospitalizations, and deaths that were reinfections were consistently higher across variant periods among adults aged 18–49 years compared with those among adults aged ≥50 years. The median interval between infections ranged from 269 to 411 days by week, with a steep decline at the start of the BA.4/BA.5 period, when >50% of reinfections occurred among persons previously infected during the Alpha variant period or later. To prevent severe COVID-19 outcomes, including those following reinfection, CDC recommends staying up to date with COVID-19 vaccination and receiving timely antiviral treatments, when eligible.*

By September 2021, approximately 150 million total SARS-CoV-2 infections were estimated to have occurred in the United States, suggesting a cumulative incidence of 44% in the population.^†^ The number of reinfections is expected to increase as the cumulative incidence of first infections rises, infection- and vaccine-induced immunity wane, and novel variants with increased transmissibility and immune escape characteristics emerge (*1*). The risk for reinfection also might vary individually based on demographic characteristics, vaccination history, and exposure risk, which are known to be interrelated (*2*,*3*). The clinical impact of reinfection remains incompletely understood. Generally, reinfections have been reported to be less clinically severe than initial SARS-CoV-2 infections (*3*,*4*); however, in some studies, reinfections were associated with severe outcomes, particularly among persons who were hospitalized with a previous infection (*4*,*5*). To describe trends over time, laboratory-confirmed SARS-CoV-2 reinfections and associated severe outcomes were characterized during a 16-month period when the Delta variant and several Omicron lineages were predominant in the United States.

Weekly, age-stratified counts of COVID-19 cases,^§^ COVID-19–associated hospitalizations,^¶^ and COVID-19–associated deaths** for all infections and reinfections occurring among adults aged ≥18 years during September 5, 2021–December 31, 2022, were reported by 18 U.S. jurisdictions. A SARS-CoV-2 reinfection was defined as a positive result^††^ from a SARS-CoV-2 RNA or antigen test performed on a respiratory specimen collected >90 days after a previous confirmed or probable COVID-19 case in the same person, based on national surveillance guidance.^§§^ Using this definition, reinfections were included if previous infections occurred during March 1, 2020–October 1, 2022. Multiple occurrences of reinfection in a person were included as separate reinfection events, provided each met the same criteria. COVID-19–associated hospitalizations^¶¶^ and deaths*** were defined by participating U.S. jurisdictions. Periods of SARS-CoV-2 variant predominance (i.e., accounting for ≥50% of circulating variants) were defined using estimated variant proportions from national genomic surveillance.^†††^

Percentages of reinfections among all COVID-19 cases, hospitalizations, and deaths were calculated by age group and variant period. Overall weekly trends in median time to reinfection (i.e., the interval from previous infection to reinfection) were estimated by weighting reported weekly medians using the number of weekly reinfections per jurisdiction. Weekly trends in median time to reinfection were compared with trends in the percentage distribution of variant predominant periods of the previous infection. R software (version 4.1.3; R Foundation) was used to conduct all analyses. This activity was reviewed by CDC and conducted consistent with applicable federal law and CDC policy.^§§§^

During September 5, 2021–December 31, 2022, a total of 2,784,548 laboratory-confirmed SARS-CoV-2 reinfections were reported among adults aged ≥18 years from 18 U.S. jurisdictions, accounting for 12.7% of all SARS-CoV-2 infections reported in this same population and period (21,943,686). Adults aged 18–49 years (who constitute 54% of the U.S. population) accounted for 66.8% of reinfections and 62.0% of overall infections during this period, whereas adults aged 50–64 years and ≥65 years accounted for 21.2% and 11.9% of reinfections, respectively. Reinfections represented 2.7% of all reported SARS-CoV-2 infections during the Delta variant period in late 2021; this percentage increased to 10.3% during the Omicron BA.1 period, 12.5% during the BA.2 period, 20.6% during the BA.4/BA.5 period, and 28.8% during the BQ.1/BQ.1.1 periods (Table) (Figure 1). The absolute increase in the percentage of reinfections among all reported SARS-CoV-2 infections was highest among adults aged 18–49 years, increasing from 3.0% during the Delta period to 34.4% during the Omicron BQ.1/BQ.1.1 period. Among adults aged 50–64 years, the percentage of reinfections among all infections increased from 2.1% (Delta) to 29.0% (Omicron BQ.1/BQ1.1), and among those aged ≥65 years, reinfections increased from 2.0% (Delta) to 18.9% (Omicron BQ.1/BQ.1.1). Among a subset of 2,008,867 persons with one or more reinfections reported by 13 jurisdictions identifying multiple reinfections,^¶¶¶^ 95.6% experienced one reinfection, 4.3% experienced two reinfections, and 0.2% experienced three or more reinfections during September 5, 2021–December 31, 2022.

**TABLE:** Reported numbers of all SARS-CoV-2 infections and numbers and percentages of reinfections,* by age group, outcome, and variant period^†^ — 18 U.S. jurisdictions,^§^ September 5, 2021–December 31, 2022

Outcome/ Age group, yrs	Sep 5–Dec 18, 2021 Delta		Dec 19, 2021–Mar 19, 2022 Omicron BA.1		Mar 20–Jun 18, 2022 Omicron BA.2		Jun 19–Nov 5, 2022 Omicron BA.4/BA.5		Nov 6–Dec 31, 2022 Omicron BQ.1/BQ.1.1		Sep 5, 2021–Dec 31, 2022 (full outcome period)	
	Reinfections (% of all infections)	All infections	Reinfections (% of all infections)	All infections	Reinfections (% of all infections)	All infections	Reinfections (% of all infections)	All infections	Reinfections (% of all infections)	All infections	Reinfections (% of all infections)	All infections
**Cases, 18 jurisdictions**												
18–49	60,818 (3.0)	2,020,296	779,482 (11.1)	7,032,087	224,417 (14.1)	1,586,446	566,806 (24.6)	2,307,711	229,271 (34.4)	666,385	1,860,794 (13.7)	**13,612,925**
50–64	14,164 (2.1)	674,988	207,143 (9.4)	2,199,892	70,852 (11.6)	611,837	198,781 (19.2)	1,033,953	100,672 (29.0)	347,292	591,612 (12.2)	**4,867,962**
≥65	8,332 (2.0)	418,111	89,281 (7.4)	1,212,266	39,932 (8.4)	477,769	121,688 (12.6)	968,243	72,909 (18.9)	386,410	332,142 (9.6)	**3,462,799**
**Overall**	**83,314 (2.7)**	**3,113,395**	**1,075,906 (10.3)**	**10,444,245**	**335,201 (12.5)**	**2,676,052**	**887,275 (20.6)**	**4,309,907**	**402,852 (28.8)**	**1,400,087**	**2,784,548 (12.7)**	**21,943,686**
**Hospitalizations, 10 jurisdictions**												
18–49	717 (2.6)	27,138	5,123 (10.8)	47,439	1,870 (17.1)	10,912	5,272 (21.3)	24,731	2,324 (24.8)	9,356	15,306 (12.8)	**119,576**
50–64	446 (1.7)	26,915	3,023 (7.7)	39,290	1,191 (14.4)	8,251	3,777 (18.6)	20,354	2,040 (22.7)	8,991	10,477 (10.1)	**103,801**
≥65	689 (1.7)	41,451	3,919 (4.7)	83,225	2,009 (7.8)	25,686	6,542 (9.9)	65,991	4,490 (13.3)	33,689	17,649 (7.1)	**250,042**
**Overall**	**1,852 (1.9)**	**95,504**	**12,065 (7.1)**	**169,954**	**5,070 (11.3)**	**44,849**	**15,591 (14.0)**	**111,076**	**8,854 (17.0)**	**52,036**	**43,432 (9.2)**	**473,419**
**Deaths, 17 jurisdictions**												
18–49	58 (1.4)	4,158	160 (4.7)	3,407	71 (16.1)	442	121 (14.0)	864	69 (20.2)	341	479 (5.2)	**9,212**
50–64	103 (1.1)	9,723	400 (3.9)	10,199	145 (13.2)	1,098	355 (14.9)	2,387	165 (15.9)	1,040	1,168 (4.8)	**24,447**
≥65	316 (1.2)	25,952	1,569 (3.6)	43,267	658 (8.9)	7,386	1,686 (9.4)	17,894	1,012 (11.6)	8,755	5,241 (5.1)	**103,254**
**Overall**	**477 (1.2)**	**39,833**	**2,129 (3.7)**	**56,873**	**874 (9.8)**	**8,926**	**2,162 (10.2)**	**21,145**	**1,246 (12.3)**	**10,136**	**6,888 (5.0)**	**136,913**

* A SARS-CoV-2 reinfection was defined as a SARS-CoV-2 RNA or antigen detection (based on confirmatory or presumptive laboratory evidence, as defined by the Council of State and Territorial Epidemiologists) on a respiratory specimen collected >90 days after a previous confirmed or probable COVID-19 case in the same person. https://ndc.services.cdc.gov/case-definitions/coronavirus-disease-2019-2021/

^†^ Periods were defined using ≥50% SARS-CoV-2 variant proportions from national genomic surveillance: ancestral strain (April 3, 2021, and earlier); Alpha (April 4–June 19, 2021); Delta (June 20–December 18, 2021); BA.1 comprising Omicron B.1.1.529 and BA.1.1 (December 19, 2021–March 19, 2022); BA.2 comprising BA.2 and BA.2.12.1 (March 20–June 18, 2022); and BA.4/BA.5 comprising BA.4, BA.4.6, and BA.5 (June 19–November 5, 2022). The BQ.1/BQ.1.1 period (November 6–December 31, 2022) also included other lineages with similar spike protein substitutions and was defined based on when BA.4/BA.4.6/BA.5 reached <50%, as these other lineages increased. https://covid.cdc.gov/covid-data-tracker/#variant-proportions

^§^ Data on COVID-19 reinfection–associated cases were included from the following 18 jurisdictions, representing 45% of the U.S. population: California, Colorado, District of Columbia, Georgia, Indiana, Kentucky, Louisiana, Massachusetts, Minnesota, Nebraska, New Jersey, New York, New York City, North Carolina, Oregon, Philadelphia, Tennessee, and Washington; of these, New York did not report data on COVID-19–associated deaths. Data on COVID-19 reinfection–associated hospitalizations were included from 10 jurisdictions: California, Colorado, Georgia, Minnesota, New Jersey, New York City, Oregon, Philadelphia, Tennessee, and Washington.

**FIGURE 1:**
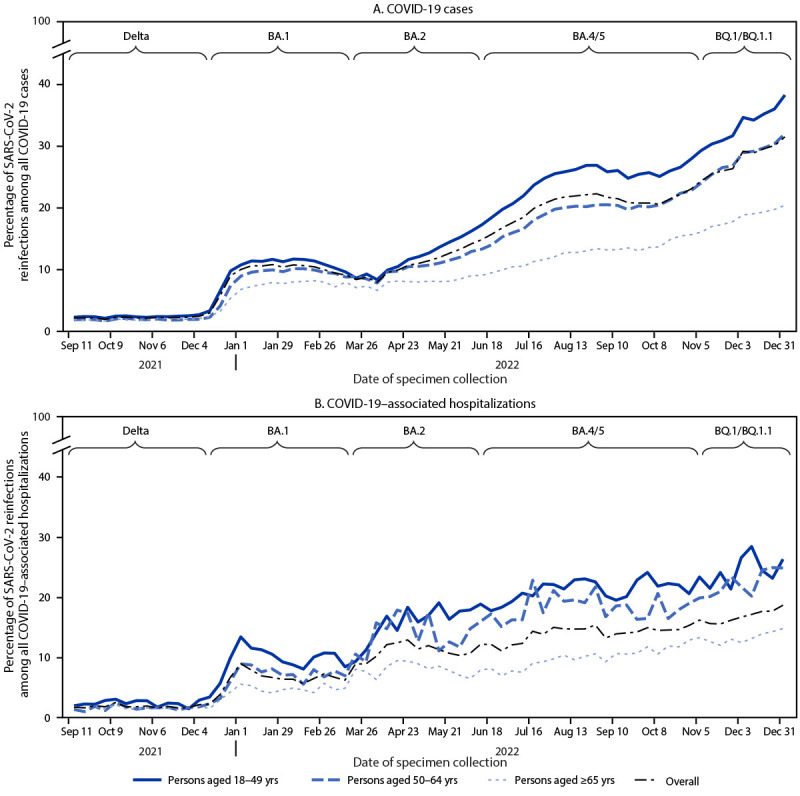
Percentages of SARS-CoV-2 reinfections* among all infections for COVID-19 cases (A) and COVID-19–associated hospitalizations (B), by week of positive specimen collection date, age group, and SARS-CoV-2 variant period^†^ — 18 U.S. jurisdictions,^§^ September 5, 2021–December 31, 2022 * A SARS-CoV-2 reinfection was defined as a SARS-CoV-2 RNA or antigen detection (based on confirmatory or presumptive laboratory evidence, as defined by the Council of State and Territorial Epidemiologists) in a respiratory specimen collected >90 days after a previous confirmed or probable COVID-19 case in the same person. https://ndc.services.cdc.gov/case-definitions/coronavirus-disease-2019-2021/ ^†^ Periods were defined using ≥50% SARS-CoV-2 variant proportions from national genomic surveillance: ancestral strain (April 3, 2021, and earlier); Alpha (April 4–June 19, 2021); Delta (June 20–December 18, 2021); BA.1 comprising Omicron B.1.1.529 and BA.1.1 (December 19, 2021–March 19, 2022); BA.2 comprising BA.2 and BA.2.12.1 (March 20–June 18, 2022); and BA.4/BA.5 comprising BA.4, BA.4.6, and BA.5 (June 19–November 5, 2022). The BQ.1/BQ.1.1 period (November 6–December 31, 2022) also included other lineages with similar spike protein substitutions and was defined based on when BA.4/BA.4.6/BA.5 lineages reached <50%, as these other lineages increased. https://covid.cdc.gov/covid-data-tracker/#variant-proportions ^§^ Data on reinfection–associated COVID-19 cases were included from 18 jurisdictions, representing 45% of the U.S. population: California, Colorado, District of Columbia, Georgia, Indiana, Kentucky, Louisiana, Massachusetts, Minnesota, Nebraska, New Jersey, New York, New York City, North Carolina, Oregon, Philadelphia, Tennessee, and Washington. Data on COVID-19 reinfection-associated hospitalizations were included from 10 jurisdictions: California, Colorado, Georgia, Minnesota, New Jersey, New York City, Oregon, Philadelphia, Tennessee, and Washington.

Among SARS-CoV-2 reinfections, 43,432 associated hospitalizations and 6,888 associated deaths were reported from 10 and 17 U.S. jurisdictions, respectively. Increases in the percentages of reinfections among reported COVID-19–associated hospitalizations and deaths were similar to those for COVID-19 cases but with decreased magnitude. The percentages of reported reinfections among COVID-19–associated hospitalizations and deaths increased substantially from 1.9% and 1.2%, respectively, during the Delta period, to 17.0% and 12.3%, respectively, during the Omicron BQ.1/BQ.1.1 period (Table) (Figure 1) (Supplementary Figure, https://stacks.cdc.gov/view/cdc/129923). Among COVID-19–associated hospitalizations and deaths, reinfections were more prevalent among adults aged 18–49 years, compared with older adults, especially during late 2022. Reinfections accounted for 24.8% of hospitalizations and 20.2% of deaths in adults aged 18–49 years during the BQ.1/BQ.1.1 period; by comparison, reinfections accounted for 13.3% of hospitalizations and 11.6% of deaths among adults aged ≥65 years during this period.

Among 17 reporting jurisdictions,**** the median interval between infections by week increased from 269 days in September 2021 to a peak of 411 days in mid-February 2022, near the end of the BA.1 period (Figure 2). The median time to reinfection decreased substantially to 335 days in mid-June 2022 after the start of the BA.4/BA.5 period and remained near that level for the remainder of BA.4/BA.5 predominance. By the week ending December 31, 2022 (the BQ.1/BQ.1.1 period), the median time to reinfection had increased to 367 days.

**FIGURE 2:**
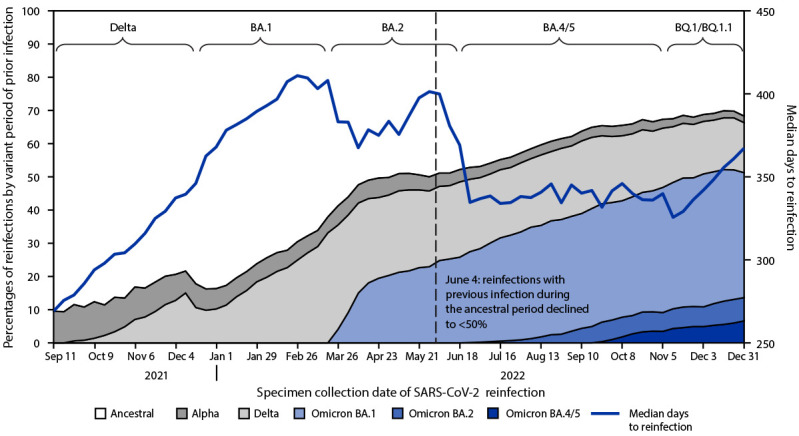
Weekly proportions of SARS-CoV-2 reinfections,* by variant period^†^ of the previous infection and median time^§^ to reinfection — 17 U.S. jurisdictions,^¶^ September 5, 2021–December 31, 2022 * A SARS-CoV-2 reinfection was defined as SARS-CoV-2 RNA or antigen detection (based on confirmatory or presumptive laboratory evidence, as defined by the Council of State and Territorial Epidemiologists) on a respiratory specimen collected >90 days after a previous confirmed or probable COVID-19 case in the same person. https://ndc.services.cdc.gov/case-definitions/coronavirus-disease-2019-2021/ ^†^ Periods of previous infections and reinfections were defined using ≥50% SARS-CoV-2 variant proportions from national genomic surveillance: ancestral strain (April 3, 2021, and earlier); Alpha (April 4–June 19, 2021); Delta (June 20–December 18, 2021); BA.1 comprising Omicron B.1.1.529 and BA.1.1 (December 19, 2021–March 19, 2022); BA.2 comprising BA.2 and BA.2.12.1 (March 20–June 18, 2022); and BA.4/BA.5 comprising BA.4, BA.4.6, and BA.5 (June 19–November 5, 2022). The BQ.1/BQ.1.1 period (November 6–December 31, 2022) also included other lineages with similar spike protein substitutions and was defined based on when BA.4/BA.4.6/BA.5 lineages reached <50%, as these other lineages increased. https://covid.cdc.gov/covid-data-tracker/#variant-proportions ^§^ Overall weekly trends in the median time to reinfection (i.e., median days between positive specimen collection dates) were estimated by weighting the reported medians using the number of weekly reinfections per jurisdiction. ^¶^ Data were included for 17 jurisdictions: California, Colorado, District of Columbia, Georgia, Indiana, Kentucky, Louisiana, Massachusetts, Minnesota, Nebraska, New Jersey, New York, New York City, North Carolina, Oregon, Philadelphia, and Washington.

Among persons reinfected in September 2021, 90.5% had been previously infected during the period when the ancestral strain was predominant, and 9.5% had been previously infected during the Alpha variant period (Figure 2). The large decline in weekly median time to reinfection in June 2022 (at the transition from BA.2 to BA.4/BA.5 predominance) occurred when the proportion of persons previously infected during the ancestral period declined to <50%; conversely, the proportion previously infected during more recent variant periods (i.e., Alpha, Delta, or Omicron) increased to >50%. By the end of 2022, during the Omicron BQ.1/BQ.1.1 period, 51.3% of reinfected persons had been previously infected earlier in the Omicron period (BA.1 = 37.6%; BA.2 = 7.0%; and BA.4/BA.5 = 6.6%), with the remainder having been previously infected during periods when the ancestral strain (31.7%), Delta variant (15.0%), or Alpha variant (2.0%) were predominant.

## Discussion

This descriptive analysis of surveillance data reported by 18 jurisdictions shows that cases of SARS-CoV-2 reinfection and associated hospitalizations and deaths increased in relative frequency as new Omicron lineages emerged with enhanced transmissibility or immune escape characteristics^††††^ (*1*), and as the number of persons with first infections increased over time. The weekly median time between infections ranged from 269 to 411 days, with a steep drop observed at the start of the BA.4/BA.5 period, when >50% of reinfections occurred among persons previously infected during the Alpha variant period or later. The changing distribution of variants associated with previous SARS-CoV-2 infections and reinfections over time mirrors observations reported from other studies (*1*,*4*) and highlights the increasing complexity of the SARS-CoV-2 immunologic landscape (*6*).

Higher percentages of reinfections among COVID-19 cases and associated hospitalizations and deaths were observed among younger adults compared with older adults, particularly in late 2022. The higher percentages in younger age groups might be attributable to multiple factors, including higher cumulative incidence of first infections, later eligibility for vaccination, lower vaccination coverage, increased exposure risk, and a possible survival bias because of less severe initial infections (*6*). Reinfections occurred at lower frequencies among persons who were hospitalized or died compared with cases,^§§§§^ consistent with evidence that previous infection-induced immunity provides better protection against severe outcomes than against subsequent infections (*7*). The risk of severe outcomes from reinfection can be reduced through vaccination (*7*,*8*), although vaccine effectiveness was not evaluated in this analysis.

The findings from this report are subject to at least six limitations. First, cases of COVID-19 might be increasingly underascertained by public health surveillance because of increasing use of at-home tests throughout 2022 (*9*). Reinfections might not be captured by surveillance if either previous infections or reinfections are not laboratory-confirmed or cannot be linked (e.g., laboratory-confirmed in different jurisdictions). Second, trends in reinfections before September 1, 2021, were not determined because of the lack of a nationally standardized surveillance definition for reinfection before that time. Third, the use of the 90-day definition for reinfections based on national guidance excludes reinfections occurring ≤90 days, which would need to be confirmed using genomic sequencing to rule out prolonged viral shedding.^¶¶¶¶^ Fourth, a subset of the 18 jurisdictions submitted data on reinfection-associated severe outcomes, and definitions and approaches used for ascertaining COVID-19–associated hospitalizations and deaths varied by jurisdiction. Fifth, this ecologic analysis of epidemiologic changes in reinfection by period of SARS-CoV-2 variant predominance could not adjust for important confounders, including changes in immunity, behavior, and the population at risk over time (*6*). Finally, this descriptive analysis did not determine the impact of vaccination because it was not possible to adjust for confounding differences in testing behaviors or underlying health conditions by vaccination status.

Some data sources used for this analysis, including test results from electronic laboratory reporting data, have changed or have been discontinued with the expiration of the public health emergency declaration on May 11, 2023 (*10*). However, continued monitoring of reinfections using alternative data sources remains important to characterize trends in severe outcomes following reinfection. To reduce the risk for severe COVID-19–associated outcomes, including those after reinfection, CDC recommends staying up to date with COVID-19 vaccination***** and receiving early antiviral treatment, when eligible.

**Summary**

**What is already known about this topic?**

Although SARS-CoV-2 reinfections have increased, U.S. epidemiologic trends and associated severe outcomes have not been characterized.

**What is added by this report?**

During September 2021–December 2022, the percentages of reinfections among all COVID-19 cases, hospitalizations, and deaths reported by 18 U.S. jurisdictions increased substantially as new Omicron lineages became predominant. Increases were more pronounced among adults aged 18–49 years compared with those among older persons.

**What are the implications for public health practice?**

Cases and severe outcomes associated with SARS-CoV-2 reinfection have increased across the United States since September 2021. CDC recommends staying up to date with COVID-19 vaccinations and receiving early antiviral treatment, if eligible, to reduce the risk for severe COVID-19–associated outcomes.

## Footnotes

*
www.cdc.gov/coronavirus/2019-ncov/your-health/reinfection.html

†
https://www.cdc.gov/coronavirus/2019-ncov/cases-updates/burden.html

§ Data on SARS-CoV-2 infections and reinfections (confirmed or probable COVID-19 cases) were included for 18 jurisdictions, representing 45% of the U.S. population: California, Colorado, District of Columbia, Georgia, Indiana, Kentucky, Louisiana, Massachusetts, Minnesota, Nebraska, New Jersey, New York, New York City, North Carolina, Oregon, Philadelphia, Tennessee, and Washington.

¶ Data on COVID-19–associated hospitalizations were included for 10 jurisdictions: California, Colorado, Georgia, Minnesota, New Jersey, New York City, Oregon, Philadelphia, Tennessee, and Washington.

** Data on COVID-19–associated deaths were included for 17 jurisdictions: California, Colorado, District of Columbia, Georgia, Indiana, Kentucky, Louisiana, Massachusetts, Minnesota, Nebraska, New Jersey, New York City, North Carolina, Oregon, Philadelphia, Tennessee, and Washington.

†† Based on confirmatory or presumptive laboratory evidence as defined by the Council of State and Territorial Epidemiologists. https://ndc.services.cdc.gov/case-definitions/coronavirus-disease-2019-covid-19/

§§
https://ndc.services.cdc.gov/case-definitions/coronavirus-disease-2019-2021/

¶¶ To ascertain COVID-19–associated hospitalization, five jurisdictions relied upon hospital records and five relied upon hospital records and case investigations. Two jurisdictions reported hospitalizations only when COVID-19 was the cause. The remaining eight reported hospitalizations when a SARS-CoV-2 test result was positive within a specific time window of hospital admission, beginning 7 days (one jurisdiction), 14 days (five), 30 days (one), or 90 days (one) before admission, and lasting until 2 days (one), 7 days (one), 14 days (three), or 21 days (one) after admission, or for the duration of hospitalization (two).

*** To determine COVID-19–associated deaths, 10 jurisdictions relied upon vital records and seven relied upon both vital records and provider reporting or case investigations. Six jurisdictions provided only deaths with COVID-19 as a listed underlying or probable cause. The remaining 11 jurisdictions also provided deaths from natural causes or without other evident causes that occurred ≤30 days of positive specimen collection (eight) or some other specified time window (42 days, 45 days, and 60 days).

††† Periods were defined using ≥50% SARS-CoV-2 variant proportions from national genomic surveillance: ancestral strain (April 3, 2021 and earlier); Alpha (April 4–June 19, 2021); Delta (June 20–December 18, 2021); BA.1 comprising Omicron B.1.1.529/BA.1.1 (December 19, 2021–March 19, 2022); BA.2 comprising BA.2/BA.2.12.1 (March 20–June 18, 2022); and BA.4/BA.5 comprising BA.4/BA.4.6/BA.5 (June 19–November 5, 2022). The BQ.1/BQ.1.1 period (November 6–December 31, 2022) also included other lineages with similar spike protein substitutions and was defined based on when BA.4/BA.4.6/BA.5 lineages reached <50%, as these other lineages increased. https://covid.cdc.gov/covid-data-tracker/#variant-proportions

§§§ 45 C.F.R. part 46.102(l)(2), 21 C.F.R. part 56; 42 U.S.C. Sect. 241(d); 5 U.S.C. Sect.552a; 44 U.S.C. Sect. 3501 et seq.

¶¶¶ Data on counts of persons with multiple reinfections were included for 13 jurisdictions: Georgia, Indiana, Kentucky, Louisiana, Massachusetts, Michigan, Minnesota, Nebraska, New York, New York City, Oregon, Philadelphia, and Washington.

**** Data on median time between infections were included for 17 jurisdictions: California, Colorado, District of Columbia, Georgia, Indiana, Kentucky, Louisiana, Massachusetts, Minnesota, Nebraska, New Jersey, New York, New York City, North Carolina, Oregon, Philadelphia, and Washington.

†††† https://www.cdc.gov/coronavirus/2019-ncov/science/science-briefs/scientific-brief-omicron-variant.html; https://www.cdc.gov/mmwr/volumes/72/wr/mm7224a2.htm?s_cid=mm7224a2_w

§§§§ Trends were similar after limiting this comparison to jurisdictions that reported data on all three outcomes (COVID-19 reinfection percentages for cases, hospitalizations, and deaths).

¶¶¶¶ https://wwwnc.cdc.gov/eid/article/28/11/22-1109_article; https://www.cdc.gov/mmwr/volumes/71/wr/mm7114a2.htm

*****
https://www.cdc.gov/coronavirus/2019-ncov/vaccines/stay-up-to-date.html
